# Supplementary material for: A Boolean gene regulatory model of heterosis and speciation
Source: BMC Evol Biol. 2015 Feb 24;15:24. doi: 10.1186/s12862-015-0298-0 (PMC4349475; doi:10.1186/s12862-015-0298-0)
Supplement: Additional file 1: — Text S1. Examples of heterosis by different mechanisms. Text S2. Calculating network fitness. Text S3. Simulation results using two environments. Text S4. Generating alleles and building diploid networks. Text S5. Implementation of mutations. Text S6. Implementation of independent assortment. Text S7. Implementation of selection. Text S8. Synchronous versus asynchronous updating. [file 12862_2015_298_MOESM1_ESM.pdf]

A Boolean Gene Regulatory Model of Heterosis and Speciation

Peter M. F. Emmrich, Hannah E. Roberts, Vera Pancaldi

Supplementary Material

Text S1 Examples of heterosis by different mechanisms

Heterosis by local heterozygosity mechanisms

In our algorithm, we consider the effects of dominance and over-dominance at individual loci.

Consider the following example: two parent lines of a crop plant are crossed. Parent 1 is homozygous for a dominant allele that improves the frost tolerance and is susceptible to an insect herbivore that occurs in summer, while Parent 2 carries a dominant resistance allele against the herbivore but carries a non-functional recessive allele at the frost-tolerance locus. Compared to a line with neither of these alleles Parent 1 yields 20% better while Parent 2 yields 50% better. The F1 hybrid between the two lines carries one dominant allele at each locus. Compared to the all-recessive line, the hybrid yields 80% better (1.2 * 1.5 = 1.8) and thus outperforms both parent lines. These are simply additive effects, because there is no interaction between the two effects, so epistasis does not come into play here. Our algorithm would calculate the effect of dominance as follows:

	Frost tolerance	Herbivore resistance	Total
Fitness of the complete hybrid	180%	180%	180%
Fitness of hybrid without the allele from Parent 1<br><br>Fitness of hybrid without the allele from Parent 2	150%<br><br>180% *	180% *<br><br>120%	150%<br>(= Fitness of Parent 2)<br><br>120%<br>(= Fitness of Parent 1)

Average (mid-parent value)<br><br>Dominance effect at this locus	165%<br><br>+ 15%	150%<br><br>+ 30%	135%<br><br>+ 45%

Table S1: Example calculation of dominance effects at individual loci. The effects at the two loci are added together to give the contribution of dominance to the fitness of the hybrid. * in an example of over-dominance, these values would be lower than the fitness of the complete hybrid.

An example of heterosis over-dominance would be the following scenario: There is one locus of resistance genes against insect herbivores, but the allele carried homozygously by Parent 1 provides resistance against a different species of herbivore than the allele of Parent 2. The hybrid is resistant against both species of herbivores, and thus performs better than both parents. The calculation of the over-dominance effect is exactly the same as with dominance (see Table S1). A notable difference is that it is possible to achieve a hybrid performing better than both parents with just a single over-dominant locus.

Heterosis by positive epistasis

As an example of positive epistasis consider an example in which in the plants can also be affected by a fungal disease that affects tissues damaged either by frost or by herbivory. Both parents would be affected by this fungus, but the hybrid would be immune. This would be an example of positive epistasis, which contributes to the heterosis on top of the effect of dominance at the two alleles.

Adverse effects (hybrid disadvantage)

Under-dominance and epistatic incompatibility can cause a hybrid to perform worse than both of the parents. They are calculated the same as above, but lead to negative values for their fitness effects.

Under-dominance would be present if, for example, the presence of two different resistance alleles at one locus causes the plant to activate its defense response in absence of the herbivore, which would reduce fitness.

Epistatic incompatibility is very similar to under-dominance, except that the two alleles causing the reduced hybrid fitness are of different genes.

In contrast, an example of negative epistasis would be if the two parents carried complete resistance alleles against the same herbivore, but at different loci. Both parents and the hybrid would be resistant against the herbivore and would have an advantage compared to a susceptible line. However, the fitness of the hybrid is not better or worse than either of the parents. Thus, negative epistasis does not contribute to heterosis, but instead prevents the effects of local heterozygosity mechanisms from adding up.

Text S2 Calculating network fitness

To measure fitness, the attractors of the network were calculated using synchronous updating (see Discussion). The fitness under one environment was calculated by averaging the states of all nodes in the module associated with that environment (giving a value between 0 and 1) over the cycles of states of all attractors of the network. Equally, average values were calculated for the other modules. The fitness value for one environment was then given by the average state of the associated module minus the average of the averages of the other modules. Thus, each module has the same weighting in the calculation of the fitness value, regardless of how many nodes it contains. The network fitness, F_{net}, is the average of all the environment fitness values.

If we define the following sets:

$$M_{kl} = \{m_{kl1}, m_{kl2}, m_{kl3}, \dots m_{kln}\}$$

$$W_k = \{\overline{M_{k1}}, \overline{M_{k2}}, \overline{M_{k3}}, \dots \overline{M_{kenv}}\}$$

where:

env	Number of environments (also the number of modules)
M_{kl}	set of states of the nodes in the lth module in the kth environment,
n	number of nodes in the lth module and
W_k	set of average states of all modules

then the network fitness F_{net} is given by:

$$F_{net} = \frac{\sum_{k=1}^{env} \overline{M_{kk}} - (\overline{W_k - \overline{M_{kk}}})}{env}$$

This results in a fitness value between 1, for a perfectly responding network, and -1, for a network that responds in exactly the wrong way to every environment). In naive, un-adapted networks that serve as the ancestors for the evolutionary simulation, the state of each node was random with respect to the environment. Since the definition of environments and modules is arbitrary, the fitness of naive networks was around zero.

Text S3 Simulation results using two environments

We repeated the simulation using all the same parameters as described in the main text, but only selected for the responses to two environments instead of three. Still, each network contained three environment nodes, but only two sets of externally assigned states were applied and the resulting regulatory responses measured. Both the shape of the adaptation curve (see figure S3A), magnitude of heterosis (when it occurred) and the relative strength of local and epistatic heterosis mechanisms (see figure S3B) were broadly similar to the results described in the main text. However, two notable differences were observed. Firstly networks gained a higher overall fitness value gained during evolution. This is unsurprising, as selecting for the responses to only two environments will cause less frustration in the system than selecting for the responses to three environments, making the evolution of well-adapted networks more likely in the former case. Secondly heterosis occurred in fewer runs of the simulation (6 out of 13) than when using three environments (11 out of 16). However, more data would have to be collected to ascertain whether this difference is significant.

A

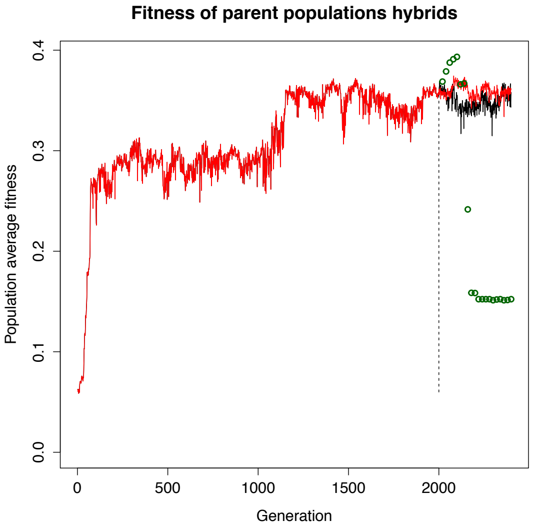

B

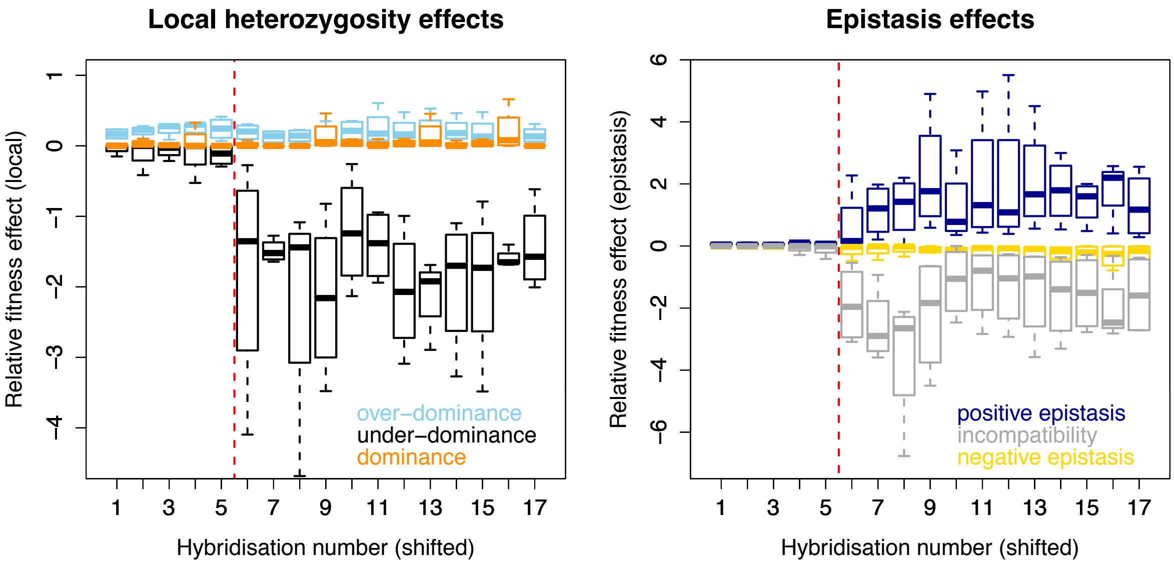

Figure S3: (A) Adaptation curve of an example population selected for its responses to two environments. The population quickly reaches a high fitness value. After the separation of the population into two populations evolving in parallel (red and black lines), both are outperformed by the inter-population hybrids (green circles) until the collapse in hybrid fitness around generation 2130. (B) Relative effects of locus heterozygosity on the fitness of hybrids resulting from selection for responses to two environments. Data of 4 runs is pooled. Data from each run was shifted so the fitness collapse coincides after the 5th hybridization (red dashed line). A) Effect of local heterozygosity mechanisms. B)

Effect of epistatic mechanisms. Please note that only gene pairs were considered for epistasis, allowing pseudoduplication of effects in cases of epistasis involving several genes. For this reason, epistatic and local heterozygosity mechanisms cannot be directly compared.

Text S4 Generating alleles and building diploid networks

We wished for the model to simulate the evolution of diploid organisms. Hence the storage of data containing information about single, haploid networks or 'gametes' was designed to allow the hybridization of two gamete networks. The following set up was used: Every node is part of a 'paralog group', formed of the copies of one gene from the initial network that have been generated by tandem duplication. In most cases these groups consist of just a single allele, but if there is more than one, they will always be inherited together. Each time a mutation (see below) occurs a new allele (version of the particular gene) is created and a data matrix containing details of all possible interactions between all alleles present in the population is updated. This matrix contains data on which alleles can have outputs to and inputs from. Depending on which alleles are present in one individual, the network connections are drawn according to this matrix. Diploid networks are formed by combining the haploid lists of alleles generated from two individuals that have passed selection and using the data matrix to fill in the correct edges.

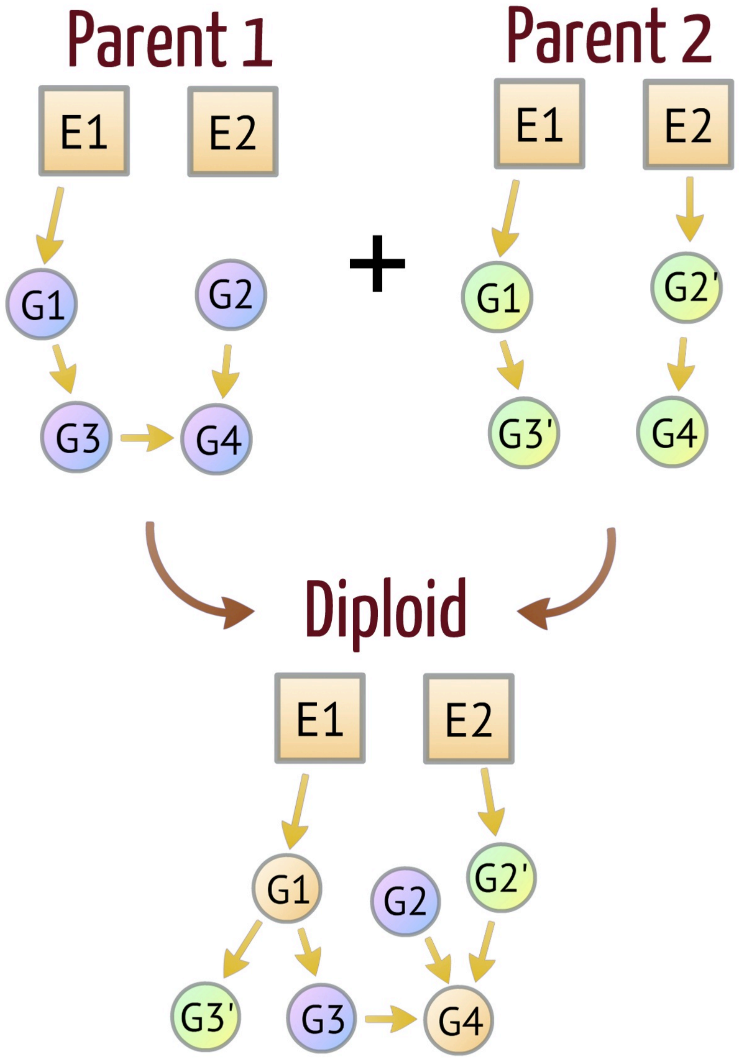

Figure S4: Example of diploid formation for an individual with four genes. The hybrid inherits identical alleles from both parents for the genes G1 and G4, but the version of G2 inherited from Parent 2 (G2') has an additional input from the environment node E2 while the version of G3 of Parent 2 (G3') lacks the ability to output to G4, as opposed to the alleles from Parent 1. In the network, each unique allele is represented by a node, so G1 and G4 appear only once. In the logic function assigning the state of G4, the two nodes G2 and G2' are linked by an OR-gate. For the hybrid this means that the node G4 could respond to inputs from both E1 (via G1 and G3) and E2 (via G2'), which could produce a fitness advantage compared to both parents.

Text S5 Implementation of mutations:

Mutation functions were designed to reflect physiological mutations such as the loss of a

binding site or the duplication of a gene (Crombach 2008; Knight and Pinney 2009). The edge mutations allow for addition and removal of ingoing and outgoing connections. Mutation functions for node duplication and loss were also included. In each generation, every individual could be subject to up to one mutation. Most individuals, however, were handed down to the next generation without any changes; the overall ratio of mutation to no mutation was kept low in order to be realistic, whilst still allowing divergence of populations in reasonable time. It has been estimated that the ratio of edge mutations to node duplication/loss during evolution is of the order 10^2:1 (Berg et al. 2004), and so the relative random mutation rates used in the simulation reflected this.

The relative rates of different mutations (see Figure 5S) used for the generation of the presented data were:

Edge addition	40
Input removal	20
Output removal	20
Node duplication	1
Node deletion	1
No mutation	2000

When an edge is added, the transition function of the receiving node must change to include this new input. This is done by adding an OR gate into the current logical expression randomly so that the new interaction is sufficient but not necessary to trigger downstream effects.

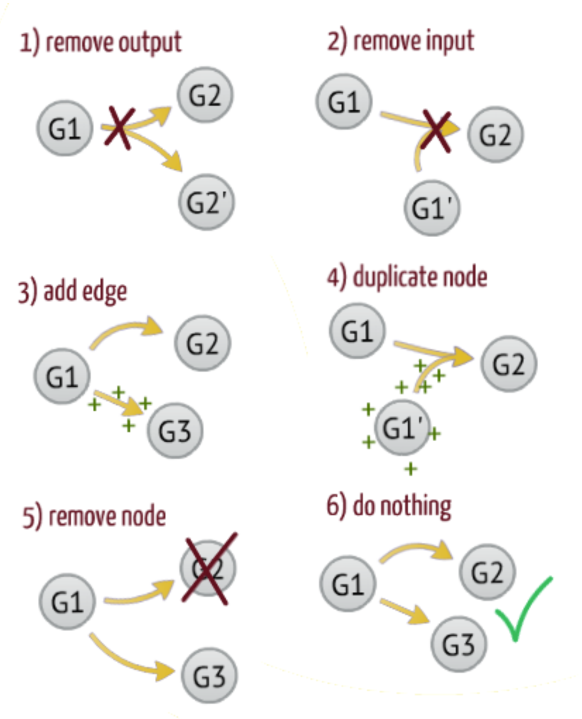

Figure S5: permitted types of mutations, which act upon haploid networks prior to the formation of the diploids of the next generation. The logic functions assigning the state of each node are changed accordingly. Rates were chosen to match biological estimates (Berg et al. 2004) In the majority of cases, the network will be handed down to the next generation without change

S6 Implementation of independent reassortment

After undergoing the mutation step, daughter haploids were formed by independently reassorting the alleles of an individual. Alleles from its two parent haploids were chosen at random and each gene was inherited from either one of the parents. If a parent had more than one allele of the same gene, these were inherited together. Beyond this, gene linkage was not considered. At this point, the population was brought back up to the size prior to selection.

S7 Implementation of selection

Networks whose offspring will form the next generation are chosen based on their overall fitness score. We wrote a Metropolis-Hastings style algorithm adapted for this particular situation. The formula takes into account the fitness of the network (F_{new}) as

well as the average fitness of the selected individuals of the previous generation (F_{old}). A network is passed on to the next generation with probability:

$$p = min\begin{cases} 1 \\ e^{-\beta(F_{old} - F_{new})} \end{cases}$$

Individuals with a higher fitness value than the previous generation will definitely be selected, but occasionally networks with lower fitness will be passed on as well. The greater the parameter β, the lower the probability that such networks survive the selection step. We empirically decided on a value of 1000 for β. This is a more realistic representation of natural selection than a simple fitness cut-off, as many random events affect the reproductive success of organisms. From the individuals that have passed selection, the parents for each individual of the next generation are chosen at random.

Text S8 Synchronous versus asynchronous updating

There are two ways of simulating the dynamics of regulatory effects in deterministic boolean networks (Faure et al. 2006). In the synchronous case, the transition functions of all nodes are evaluated based on the current state of the network. Then, the new states of all nodes are assigned. Thus, the entire network moves from one simulation step to the next in synchrony. Alternatively, the transition function of a single node can be evaluated and the new state assigned before the next node is considered. The order in which nodes are updated is chosen at random. The network dynamics are therefore asynchronous. It has been argued that asynchronous GRNs are a better representation of biology, since synchronous dynamics are liable to produce attractor artifacts (Lumer 1994). This can occur, if two nodes, which regulate each other, are assigned new states at the same time. Thus, digitalization would introduce unrealistic oscillations in the states of several nodes. Under asynchronous conditions, these oscillations would not be stable, since the order in which nodes are evaluated differs from step to step. This would lead to the asynchronous attractors being a better representation of steady states in cellular regulation in a noisy system. However, finding all attractors in asynchronous dynamics is far more computationally intensive than under the synchronous condition (Garg et al. 2008), making it unfeasible for the calculation of the attractors of large populations of networks, which would be necessary for an evolutionary algorithm. While synchronous dynamics might introduce unrealistic behavior in models of real GRNs, our simulation is only concerned with abstract networks. Any bias caused by synchronous dynamics would be applied throughout the evolutionary algorithm and to the fitness measurements of both inbred and hybrid networks. Any results

for the relative performance of hybrids compared to parents are thus likely to remain valid.

Agreement between both methods is good for small networks

Performing the calculation of fitness using both the synchronous and the asynchronous method on 1600 networks with ~20 nodes each yielded the same results in the vast majority of cases (>98.3%). This is in agreement with Gershenson's analysis, which argues that the differences between synchronous and asynchronous updating may have been exaggerated (Gershenson 2004). On the other hand, when larger networks (~40 nodes) were studied, there was a greater number of cases in which the synchronous method returned one or several attractors that were not found by the asynchronous method. These attractors are probably artifacts.

Whenever a mutation causes a new, artificial attractor to arise, it can be expected to be less well adapted than the existing ones. Thus, networks with these attractors would be selected against in the evolutionary simulation. While this introduces an unnatural factor for selection (i.e. against networks that happen to produce these artifacts), the individuals that survive are the ones in which the synchronous and the asynchronous methods return the same result. Therefore we conclude that no unreasonable bias is introduced by using the synchronous method for the calculation of network fitness.

Weighting according to basin size

If a network has more than one attractor, the fitness values measured for each attractor were weighted according to the size of the attractor basin, i.e. the number of initial states that lead into this attractor. This gives a better estimate of the network fitness than a simple average over all attractors, since attractors that are much more likely to be reached contribute more strongly than attractors that are only reached from a small number of initial states. Unfortunately, the R package BoolNet only measures basin size when using the synchronous updating method.

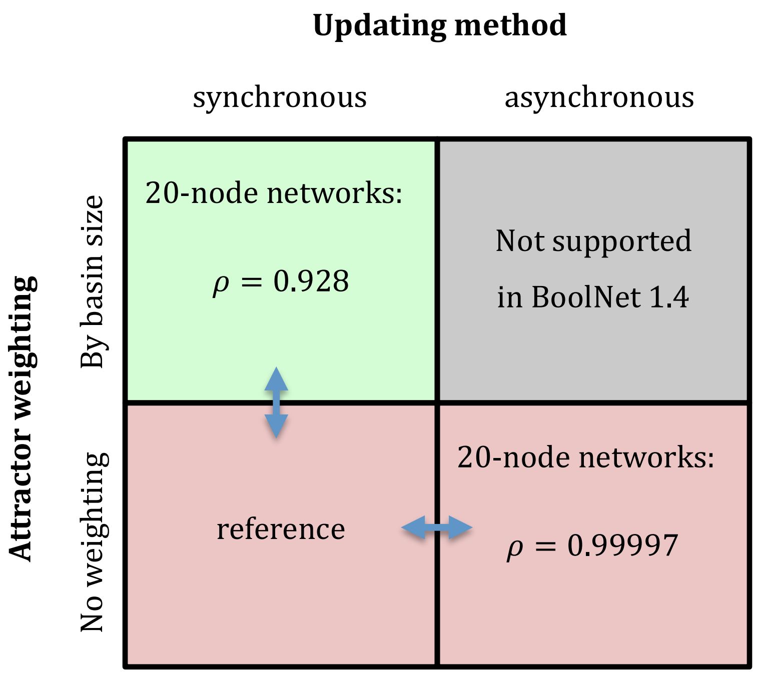

Figure S8: Pearson correlation of results gained through various methods of updating and attractor weighting. The weighted synchronous method (green) was used in this study.

When the fitness of many hybrid networks (n=2000) with around twenty nodes each was measured according to both updating methods (but with the synchronous attractors weighted according to their basin sizes), most returned the same values. This is due to the majority of networks of this size, which have undergone selection for many (>1000) generations only having one attractor, with just one state. In such an attractor, neither synchronous nor asynchronous updating cause the state of the network to change, once the steady state has been reached. Since there is only one attractor, weighting according to basin size is unnecessary. If the weighting of attractors found by the synchronous method is removed, the fitness values of heterotic hybrid networks show a very strong Pearson correlation between the two methods ($\rho = 0.99997$). Comparing the results of the synchronous updating method with and without weighting of attractors, gives a much weaker correlation ($\rho = 0.928$).

However, if larger networks (~40 nodes) were considered, most networks had more than one attractor. The basin sizes of these attractors differed widely and the correlation between fitness calculated by the synchronous and asynchronous methods (no weighting) was very weak ($\rho = 0.25$, n=800). These larger networks often contained attractors with several states that were only found when searching by the synchronous method.

The weighted synchronous method is best suited for this analysis

For larger networks, biased selection due to artificial attractors might be an issue. However, a much stronger effect is introduced if the basin size is ignored and the fitness under an environment is calculated as the simple average of the fitness values of all attractors. Since calculating basin sizes of asynchronous attractors is impossible with BoolNet, we argue that the fitness calculated by the synchronous method with appropriate weighting is the best method for the small networks (20 to 25 nodes) that were used in our simulation. For larger networks, the difference between the asynchronous and the synchronous methods, but also between weighted and non-weighted calculation are much greater, so neither method is ideal. In order to keep the computation of fitness values simple and fast, we decided to also use synchronous updating with weighted attractors for our algorithm.

References:

Berg J, Lässig M, Wagner A. 2004. Structure and evolution of protein interaction networks: a statistical model for link dynamics and gene duplications. BMC Evol. Biol. 4:51.

Crombach A. 2008. Evolution of Evolvability in Gene Regulatory Networks. PLoS Comput. Biol.

Dehmer M, Emmert-Streib F. 2011. Networks for systems biology: conceptual connection of data and function. IET Systems Biology 5:185–207.

Faure A, Naldi A, Chaouiya C, Thieffry D. 2006. Dynamical analysis of a generic Boolean model for the control of the mammalian cell cycle. Bioinformatics 22:e124–e131.

Garg A, Di Cara A, Xenarios I, Mendoza L, De Micheli G. 2008. Synchronous versus asynchronous modeling of gene regulatory networks. … 24:1917–1925.

Gershenson C. 2004. Updating Schemes in Random Boolean Networks: Do They Really Matter? Artificial Life IX Proceedings of the Ninth International Conference on the Simulation and Synthesis of Living Systems.

Knight CG, Pinney JW. 2009. Making the right connections: biological networks in the light of evolution. Bioessays 31:1080–1090.

Lumer E. 1994. ScienceDirect.com - Physica D: Nonlinear Phenomena - Synchronous versus asynchronous dynamics in spatially distributed systems. Physica D: Nonlinear Phenomena 71:440–452.
